# Omission of Sentinel Lymph Node Biopsy in Early-Stage Breast Cancer: Expanding Evidence in Clinically Node-Negative and Neoadjuvant Therapy Responders

**DOI:** 10.14740/wjon2776

**Published:** 2026-05-08

**Authors:** Abigail Grapes, Jessica Young, Kazuaki Takabe

**Affiliations:** a Breast Surgery, Department of Surgical Oncology, Roswell Park Comprehensive Cancer Center, Buffalo, NY 14203, USA; b Department of Surgery, University at Buffalo Jacobs School of Medicine and Biomedical Sciences, The State University of New York, Buffalo, NY 14203, USA; c Department of Immunology, Roswell Park Comprehensive Cancer Center, Buffalo, NY, USA; d Department of Breast Surgery and Oncology, Tokyo Medical University, Tokyo 160-8402, Japan; e Department of Gastroenterological Surgery, Yokohama City University Graduate School of Medicine, Yokohama 236-0004, Japan; f Division of Digestive and General Surgery, Niigata University Graduate School of Medical and Dental Sciences, Niigata 951-8520, Japan; g Department of Breast Surgery, Fukushima Medical University School of Medicine, Fukushima 960-1295, Japan

**Keywords:** Breast cancer, Lymph nodes, De-escalation, Omission, Sentinel lymph node biopsy, Neoadjuvant systemic therapy

## Abstract

Management of the axilla in early-stage breast cancer has progressively evolved toward less invasive approaches, as advances in tumor biology, imaging, and local and systemic therapies have improved outcomes while highlighting the morbidity associated with axillary surgery. Sentinel lymph node biopsy, once considered essential for staging, is increasingly being questioned in select patients with clinically node-negative disease. This review summarizes current evidence evaluating omission of sentinel lymph node biopsy in early-stage breast cancer, including its impact on oncologic outcomes, quality of life, and implementation in clinical practice. Multiple retrospective studies and randomized controlled trials have demonstrated that omission of axillary surgery does not compromise survival outcomes in carefully selected patients with hormone receptor (HR)-positive/human epidermal growth factor receptor 2 (HER2)-negative tumors. These findings informed the 2016 Choosing Wisely^®^ recommendation by the Society of Surgical Oncology to avoid routine sentinel lymph node biopsy in women over 70 years of age with clinically node-negative disease. While implementation of this guideline was slower than expected, subsequent studies have explored whether sentinel lymph node biopsy can be safely omitted in a broader group of women with early-stage, HR-positive/HER2-negative invasive breast cancers, who are clinically node-negative on physical exam and preoperative axillary ultrasound. This led to the development of several prospective trials, including SOUND (Sentinel Node vs Observation After Axillary Ultra-Sound), INSEMA (Intergroup Sentinel Mamma), NAUTILUS (No Axillary Surgical Treatment in Clinically Lymph Node Negative Patients on Ultrasonography After Neoadjuvant Chemotherapy), BOOG 2013-08 (Dutch Breast Cancer Research Group 2013-08), OMSLNB (Omission of Sentinel Lymph Node Biopsy), VENUS, and SOAPET (Sentinel node biopsy vs. observation after axillary PET). While many of these trials remain ongoing, results of the SOUND and INSEMA trials demonstrate no difference in survival outcomes following omission of sentinel lymph node biopsy in postmenopausal women with low grade, HR-positive/HER2-negative tumors less than 2 cm in size, and these findings are increasingly being incorporated into clinical practice. Advancements in neoadjuvant therapies have resulted in increasing rates of pathologic complete response in the breast in clinically node-negative women with HER2-positive and triple-negative breast cancers, raising the possibility that sentinel lymph node biopsy may be safely omitted in this population as well. This is being investigated in the EUBREAST-01 (European Breast Cancer Research Association of Surgical Trialists), ASICS (Avoiding Sentinel lymph node biopsy In select Clinical node negative breast cancer patients after neoadjuvant Systemic therapy), ASLAN (Avoid Axillary Sentinel Lymph Node Biopsy After Neoadjuvant Chemotherapy), and Neo-NAUTILUS trials. While these trials are ongoing, the results are promising to change the scope of current clinical practice and reduce the morbidity associated with axillary surgery in appropriately selected patients with breast cancer.

## Introduction

Management of the axilla in early-stage breast cancer has undergone a substantial paradigm shift over the past several decades, driven by advances in tumor biology, systemic therapy, and imaging that have enabled increasingly individualized and less invasive treatment strategies. Earlier detection and improvements in systemic therapy have enabled excellent locoregional control with less extensive surgery, prompting renewed scrutiny of procedures that provide limited therapeutic benefit while contributing to treatment-related morbidity. Axillary surgery has been a central focus of this de-escalation paradigm.

The Halsted radical mastectomy, involving *en bloc* removal of the breast, pectoralis muscles, and axillary contents, was historically the standard surgical procedure for breast cancer regardless of stage [1]. Although effective for local control, this approach was associated with substantial morbidity, which prompted investigation into de-escalation strategies. The landmark NSABP (National Surgical Adjuvant Breast and Bowel Project) B-04 trial challenged the therapeutic necessity of aggressive axillary surgery by randomizing patients with clinically node-negative (cN0) disease to radical mastectomy, total mastectomy with regional radiation, or total mastectomy alone [2]. After 25-year follow-up, no significant differences were observed in disease-free survival (DFS), recurrence-free survival (RFS), distant disease-free survival (DDFS), or overall survival (OS), demonstrating no survival advantage from removal of occult nodal disease or regional nodal irradiation (RNI) [3]. Subsequent trials extended surgical de-escalation to the breast. NSABP B-06 demonstrated that lumpectomy with radiation achieved survival equivalent to mastectomy in women with stage I–II disease, establishing breast conserving surgery (BCS) as standard treatment [4]. Although all patients underwent axillary lymph node dissection (ALND), this trial reinforced that increasingly conservative surgical approaches could maintain oncologic safety.

Axillary surgery is associated with well-established morbidity, including lymphedema, chronic pain, sensory deficits, shoulder dysfunction, and diminished quality of life. To minimize these risks, sentinel lymph node biopsy (SLNB), which was originally developed for melanoma, was applied to breast cancer in the 1990s, enabling accurate staging with reduced morbidity [5–9]. A series of trials then investigated whether SLNB could safely replace ALND in patients with clinically node-negative disease without compromising survival. The NSABP B-32 trial randomized clinically node-negative patients undergoing lumpectomy or mastectomy to SLNB followed by ALND versus SLNB alone and demonstrated equivalent OS, DFS, and regional control among node-negative patients treated with SLNB alone [10]. Similarly, the International Breast Cancer Study Group (IBCSG) 23-01 and AATRM 048 trials demonstrated no survival benefit from completion ALND in patients with micrometastatic nodal disease detected on SLNB [11, 12]. For patients with limited macrometastatic nodal involvement, ACOSOG Z0011 established that completion ALND (cALND) could be safely omitted in women with cT1–2N0 breast cancer and 1–2 positive sentinel lymph nodes undergoing BCS with radiation and systemic therapy, without compromising survival or regional control [13, 14]. These findings were supported by subsequent trials, including SINODAR-ONE and SENOMAC, which broadened eligibility to include mastectomy patients and continued to demonstrate oncologic safety with omission of ALND [15, 16]. The SERC trial further expanded eligibility to patients with cT0–2N0 breast cancer with any number of positive SLNs, survival outcomes remain pending [17].

In parallel, several studies evaluated the accuracy of SLNB following neoadjuvant systemic therapy (NAST). Trials including SENTINA (SENTinel NeoAdjuvant), ACOSOG Z1071, SN FNAC, and the Swedish trial demonstrated false-negative rates (FNR) exceeding the acceptable 10% threshold. However, subsequent analyses showed that accuracy improved with dual tracer mapping and retrieval of at least two sentinel lymph nodes, establishing SLNB as a feasible approach for patients following NAST [18–21]. This led to the development of the targeted axillary dissection technique to improve accuracy of axillary staging in node-positive disease following NAST.

The Cancer and Leukemia Group B (CALGB) 9343, a randomized trial evaluated adjuvant radiation after lumpectomy and tamoxifen in women ≥ 70 years old with cT1N0, estrogen receptor (ER)-positive breast cancer; although axillary surgery was not required and over 60% of patients lacked nodal staging, regional recurrence (RR) remained low [22]. At 10 years, radiation improved locoregional control (98% vs. 91%) without affecting distant metastasis, breast cancer-specific survival (BCSS), or OS, supporting safety of omitting axillary surgery in select older patients receiving endocrine therapy. In a trial by Martelli et al, women aged 60–85 years with cT1N0 breast cancer were randomized to lumpectomy with or without ALND, and all women received adjuvant radiation and tamoxifen [23]. At 15-year follow-up, there were small differences in axillary recurrence between ALND versus the no ALND arm (0% vs. 6%); however, this did not translate into differences in OS or breast cancer mortality, supporting omission of axillary surgery in this population. The IBCSG Trial 10-93 randomized women ≥ 60 years old with cT1–3N0M0 breast cancer to breast surgery (mastectomy or BCS) with or without axillary clearance, followed by 5 years of tamoxifen, and found worse early postoperative quality of life with axillary surgery, particularly with respect to arm pain and restricted mobility, but no significant differences in DFS or OS after 6 years [24]. Despite being underpowered for survival endpoints, the results supported omission of axillary surgery in clinically node-negative postmenopausal women, especially with ER-positive disease receiving endocrine therapy. Collectively, these trials formed the foundation of randomized controlled trial evidence supporting the 2016 Society of Surgical Oncology (SSO) Choosing Wisely^®^ recommendation to reduce low-value breast care, advising against routine SLNB in women ≥ 70 years of age, who are clinically node-negative with early-stage, hormone receptor (HR)-positive/human epidermal growth factor receptor 2 (HER2)-negative invasive breast cancer [25].

While implementation of this guideline was slower than expected, many questioned if SLNB could be omitted in a broader population of women with early-stage, HR-positive/HER2-negative invasive breast cancers who were clinically node-negative on physical exam and preoperative axillary ultrasound. Advancements in neoadjuvant therapies have also resulted in increasing rates of pathologic complete response (pCR) in the breast in clinically node-negative women with HER2-positive and triple-negative breast cancers (TNBC), leading to the question of whether SLNB may be safely omitted in this patient population as well. In this review, we summarize current clinical trials investigating omission of SLNB in clinically node-negative patients defined by preoperative axillary imaging, examine the impact of SLNB omission on quality of life, discuss barriers to implementation, explore deimplementation strategies, and review trials evaluating expansion of SLNB omission following NAST.

## Omission of SLNB in Women of Any Age

### Impact of omitting SLNB on survival and recurrence outcomes in women of any age

After numerous retrospective studies supported omission of axillary surgery in postmenopausal women and identified a low-risk patient population, the next question became whether this strategy could be safely extended to women across a broader age range. A prospective, observational, multicenter Swedish study followed 1,543 women (median age 61, range 30–85) with pT1a–b, grade I–II tumors after BCS or mastectomy without axillary staging between 1997 and 2002 addressed this [26]. Despite differences in contemporary treatment standards, 15-year follow-up showed a 14.4% overall recurrence rate: 7.5% local, 3.0% regional, and 3.8% distant. Locoregional recurrence was significantly lower among women who received adjuvant radiation therapy (8.3% vs. 14.5%). DFS was 78.0%, OS 77%, and BCSS 93.7% [26]. Overall, this study supported omission of axillary surgery in a carefully selected low-risk patient population.

Several randomized controlled trials were subsequently developed to evaluate omission of axillary surgery in early-stage, clinically node-negative breast cancer across wider age ranges. Key design features of these major trials are summarized in Table 1 [27–33]. The INT09/98 trial randomized women with cT1N0 breast cancer to quadrantectomy with axillary lymph node dissection (QUAD) versus quadrantectomy alone (QU) between 1998 and 2003 (n = 517) [27]. Eligible patients were 18–65 years old (mean age 52.6); tumors were largely HR-positive/HER2-negative, and < 2 cm in size. All patients received whole breast radiation (WBR) and tamoxifen; additional adjuvant therapy was based on nodal status or biologic tumor features. At 10-year follow-up, OS (93.3% vs. 91.5%), DFS (92.4% vs. 91.3%), distant metastasis (8.5% vs. 8.2%), and breast cancer mortality (6.2% vs. 6.3%) were comparable between QUAD and QU groups, none of which were statistically significant. Axillary recurrence occurred in 9.0% of QU patients (median time 30.0 months) compared to none in QUAD. In the QUAD arm, 28.7% of patients were lymph node–positive [27]. At 20 years, long-term outcomes remained similar between groups: OS (77.3% vs. 76.8%), DFS (86.9% vs. 81.5%), distant metastasis (13.2% vs. 17.6%), and breast cancer mortality (11% vs. 14.3%). Axillary recurrence in the QU arm was 10.6% (median time 35.8 months) versus 0.37% in the QUAD arm (at 154.6 months) [34]. Collectively, these results demonstrated that omission of ALND did not compromise survival outcomes, despite a modest increase in axillary recurrence.

**Table 1:** Clinical Trials Evaluating Omission of Axillary Surgery in Clinically Node-Negative Early-Stage Breast Cancer

Trial	Location	Population	Study design	Primary endpoint	Secondary endpoints	Axillary lymph node evaluation	Radiation
INT09/98 [27] (NCT01508546)	Italy	Women < 65 years old with cT1N0 randomized to quadrantectomy without axillary surgery (QU) or quadrantectomy with axillary dissection (QUAD)	Randomized, noninferiority clinical trial	OS	DFS, timing of axillary lymph node metastasis in QU arm	Clinical exam	RT to the breast without intentional inclusion of the axilla, supraclavicular, or internal mammary lymph nodes
SOUND [28] (NCT02167490)	Italy, Spain, Switzerland, Chile	Women of any age with invasive breast cancer ≤ 2 cm, cN0 undergoing BCS and RT (WBR or pBI), randomized 1:1 to SLNB or no axillary surgery	Prospective, multicenter, noninferiority phase 3 randomized clinical trial	DDFS	Cumulative incidence of distant recurrences, cumulative incidence of axillary recurrences, DFS, OS, adjuvant treatment recommendations	Pre-op axillary US	WBR with conventional fractionation, intraoperative boost (ELIOT) followed by hypofractionated course of WBR or pBI
INSEMA [29] (NCT02466737)	Germany, Austria	Women ≥ 18 years old with cT1–T2, cN0 breast cancer undergoing BCS with WBR, randomized 1:4 to undergo omission of axillary surgery or to SLNB, those randomized to SLNB found to be pN1 were randomized 1:1 to cALND or omission of cALND	Prospective, multicenter, noninferiority randomized clinical trial	IDFS	OS, locoregional DFS, ipsilateral axillary recurrence, DDFS, QOL measures, dose distribution in ipsilateral axilla levels I–III during RT	Clinical exam and pre-op axillary US	WBR with conventional fractionation or moderate hypofractionation, without targeting of the axilla, boost RT to the tumor bed recommended
BOOG2013-08 [30] (NCT02271828)	Netherlands	Women ≥ 18 years old with cT1–2N0 breast cancer undergoing BCS with WBR randomized to SLNB or no axillary surgery	Prospective, multicenter, noninferiority randomized clinical trial	RR	DDFS, OS, local recurrence rate, contralateral breast cancer, delayed axillary treatment, adjuvant RT, QOL, morbidity, cost-effectiveness	Clinical exam and pre-op axillary US	WBR
NAUTILUS [31] (NCT04303715)	Korea	Women ≥ 19 years old with cT1–2N0 breast cancer undergoing BCS with WBR, ECOG performance status 0–2, randomized 1:1 to SLNB or no axillary surgery	Prospective, multicenter, noninferiority randomized clinical trial	IDFS	OS, distant metastasis free survival, local recurrence, axillary recurrence, QOL	Clinical exam and pre-op axillary US	WBR for the no-SLNB arm, inclusion of axillary levels I and II are recommended within the tangential field
OMSLNB [33] (NCT05935150)	China	Women 18–70 years old with breast cancers ≤ 3 cm, cN0 undergoing BCS with WBR or mastectomy without axillary surgery, with ECOG performance status 0–1	Prospective, single-arm, noninferiority, phase II, open-label trial	IDFS	Local recurrence, regional recurrence, locoregional recurrence, incidence of lymphedema, QOL	Clinical exam and two or more imaging tests, including axillary US and MRI, PET-CT, or 18F-FDG PET/MRI	WBR (excluding the axilla)
VENUS [32] (NCT05315154)	Brazil	Women ≥ 18 years old with cT1–2N0 breast cancer undergoing BCS or mastectomy, randomized to SLNB or no axillary surgery. Women undergoing neoadjuvant systemic therapy were not excluded.	Prospective, noninferiority, randomized clinical trial	DFS	OS, regional recurrence-free survival, axillary recurrence rate, axillary morbidity, US accuracy, cost effectiveness	Clinical exam and pre-op axillary US	Not specified

SLNB: sentinel lymph node biopsy; QUAD: quadrantectomy with axillary lymph node dissection; QU: quadrantectomy alone; WBR: whole breast radiation; SOUND: Sentinel Node vs Observation After Axillary Ultra-Sound; INSEMA: Intergroup Sentinel Mamma; pBI: partial breast irradiation; BOOG 2013-08: Dutch Breast Cancer Research Group 2013-08; NAUTILUS: No Axillary Surgical Treatment for Lymph Node-Negative Patients after Ultra-Sonography; OMSLNB: Omission of Sentinel Lymph Node Biopsy; MRI: magnetic resonance imaging; pCR: pathologic complete response; US: ultrasonography; BCS: breast conserving surgery; IDFS: invasive disease-free survival; OS: overall survival; DFS: disease-free survival; DDFS: distant disease-free survival; RNI: regional nodal irradiation; RT: radiotherapy; PET/CT: positron emission tomography/computed tomography; QOL: quality of life; 18F-FDG: fluorine-18 fluorodeoxyglucose; cALND: completion axillary lymph node dissection.

The SOUND (Sentinel Node vs Observation After Axillary Ultra-Sound) trial is a multicenter trial designed to demonstrate noninferiority of omission of SLNB following a negative preoperative axillary ultrasound. Women ≥ 18 years with invasive breast cancer ≤ 2 cm undergoing BCS with adjuvant radiation (including partial breast radiation) were randomized to SLNB or no axillary surgery between 2012 and 2017 [28]. Both treatment arms had high rates of adjuvant endocrine and radiation therapy. Among 1,405 analyzed patients (median age 60), 87.8% had HR-positive/HER2-negative disease, and median tumor size was 1.1 cm (interquartile range of 0.8–1.5 cm). Positive SLNB occurred in 13.7% of the SLNB arm. At a median follow-up of 5.7 years, outcomes were comparable: 5-year DDFS (97.7% vs. 98.0%), DFS (94.7% vs. 93.9%), OS (98.2% vs. 98.4%), and axillary recurrence (0.4% vs. 0.4%) in the SLNB versus observation groups, respectively [28]. Omission of SLNB was therefore non-inferior in this population, though longer follow-up is required, with 10-year results expected in 2028.

The INSEMA (Intergroup Sentinel Mamma) trial is the largest study to date evaluating omission of axillary surgery [29]. This multicenter, prospective, noninferiority trial enrolled 4,858 women ≥ 18 years old with cT1–2, clinically node-negative (on physical exam and imaging) invasive breast cancer undergoing breast conserving surgery with WBR between 2015 and 2019. Patients were randomized 1:4 to no axillary surgery or SLNB; those with 1–3 macrometastatic sentinel lymph nodes were further randomized to cALND versus SLNB alone. Median age was 62.0, median tumor size 1.1 cm (interquartile range of 0.8–1.6 cm), and median follow-up 73.6 months. SLNB positivity occurred in 15% of patients; of those undergoing ALND, 66.8% had 1–3 positive nodes, and 13.0% had ≥ 4 positive nodes. The 5-year invasive disease-free survival (IDFS) was 91.9% vs. 91.7%, and OS was 98.2% vs. 96.9% in the omission vs SLNB groups, respectively. Axillary recurrence was nominally higher without SLNB (1.0% vs. 0.3%), but this did not affect DFS or OS. Notably, radiotherapy plan reviews showed that 50% of the 276 patients who were included in planned review of radiotherapy protocols received ≥ 80% of the breast radiation dose prescribed for the breast covering axillary level I, which may have contributed to excellent axillary control. These findings support omission of SLNB, particularly in women ≥ 50 years with cT1, grade 1–2, HR-positive/HER2-negative invasive breast cancers [29]. Preliminary results from the second randomization of the INSEMA trial—comparing cALND versus SLNB—were presented by Toralf Reimer et al [35] at the San Antonio Breast Cancer Symposium in December 2025, further supporting findings from the ACOSOG Z0011 trial. Among 386 patients in the per-protocol population, baseline tumor characteristics were well-balanced between the SLNB and cALND arms. At 5 years, IDFS was 86.6% for the SLNB arm versus 93.8% for cALND arm (hazard ratio, 1.69; 95% confidence interval (CI), 0.98–2.94), exceeding the predefined noninferiority margin, and thus noninferiority was not demonstrated. Rates of recurrence and survival were similar between arms, including local recurrence (LR; 1.4% vs. 1.2%), axillary recurrence (0.5% vs. 0%), distant metastasis (6.9% vs. 4.1%), and OS (94.9% vs. 96.2%), with a hazard ratio of 1.19 (95% CI, 0.55–2.56) for SLNB arm compared to the cALND arm. There were no significant differences in adjuvant systemic therapies between treatment arms, despite a trend toward higher rates of chemotherapy for cALND. And as one would expect, cALND had higher rates of RNI compared to the SLNB arm. Follow-up is ongoing, with 10-year results expected in 2029 [35].

Although both SOUND and INSEMA trials demonstrated short-term noninferiority of SLNB omission, they differ in key respects. SOUND included only invasive tumors ≤ 2 cm, whereas INSEMA permitted tumors ≤ 5 cm, yet both studies had a median tumor size of 1.1 cm. SOUND patients had a narrower age distribution (median 60 years, range 52–68) compared with INSEMA (median age 62 years, range 24–89). Both trials included predominantly ER/progesterone receptor (PR)+, HER2– patients. Radiation approaches differed as well. SOUND used whole and partial breast irradiation (pBI) (10.7% vs. 10.8% in SLNB vs. omission group, respectively for pBI), while INSEMA only allowed WBR and included protocol oversight revealing incidental coverage of axillary level I. Both trials included invasive ductal and lobular histology; however, SOUND also included tubular histology. Primary endpoints differed (SOUND: DDFS; INSEMA: IDFS), yet both trials support noninferiority of SLNB omission in selected early-stage, clinically node-negative cancers. Ten-year results are eagerly awaited.

Several additional prospective trials are underway to define the safety of SLNB omission across broader populations. The BOOG 2013-08 (Dutch Breast Cancer Research Group 2013-08) trial is a Dutch multicenter noninferiority study enrolling women aged ≥ 18 years with cT1–2, clinically node-negative (based on physical exam and preoperative axillary ultrasound) invasive breast cancer undergoing BCS with WBR, randomized to SLNB versus no SLNB [30]. Primary endpoints are 5- and 10-year RR; secondary outcomes include regional recurrence-free survival (RRFS), delayed axillary treatment, DDFS, OS, LR, contralateral breast cancer, axillary morbidity, radiation administration, quality of life, and cost effectiveness. The protocol estimated a sample size of 1,644 participants [30]. Three-year quality of life outcomes are available, and preliminary median 5-year follow-up data was recently presented by Smidt et al at the San Antonio Breast Cancer Symposium in December 2025 [36]. A total of 1,572 women were included in the trial, average age was 61 years old (61.4 in the SLNB arm vs. 61.6 in the no axillary surgery arm), with 89% of patients over 50 years old. Tumors were predominantly cT1 (82.1% vs. 83.7%), grade 1–2 (82.9%), HR-positive, HER2-negative (86.4% vs. 87.9%), and invasive ductal carcinoma (77.9% vs. 77.0%). Among patients undergoing SLNB, 13.7% had node-positive disease. Use of adjuvant systemic therapy was similar between groups, including chemotherapy (13.0% vs. 11.3%), targeted therapy (5.9% vs. 4.4%), and endocrine therapy (44.8% vs. 43.1%). At a median follow-up of 5 years, RRFS was 96.6% in the SLNB group and 94.2% in the no axillary surgery group, corresponding to an absolute difference of 2.35% (95% CI, 0.06–4.72), which did not exceed the 5% noninferiority margin. DDFS was 96.0% SLNB vs. 92.9% in the no axillary surgery arm, with absolute difference of 3.3%. Overall recurrence events included LR (15.3% vs. 9.1%), RR (5.1% vs. 9.1%), and distant metastasis (23.7% vs. 29.5%) for SLNB versus no axillary surgery, respectively. These preliminary findings demonstrated that omission of SLNB was non-inferior to the SLNB group for RRFS, supporting the potential feasibility of omitting SLNB in women ≥ 50 years old with HR-positive/HER2-negative, grade 1–2, T1 breast cancer [36].

The NAUTILUS (No Axillary Surgical Treatment for Lymph Node-Negative Patients after Ultra-Sonography) trial in Korea is a multicenter, randomized controlled trial enrolling women ≥ 19 years old with invasive breast cancer < 5 cm, clinically node-negative on axillary ultrasound, treated with BCS and WBR [31]. Patients are randomized to SLNB versus no SLNB. Women must have an Eastern Cooperative Oncology Group (ECOG) performance class of 0–2. Protocol guidelines recommend inclusion of ipsilateral axillary levels I–II in tangential fields of radiation for the no-SLNB arm. Primary outcome is 5-year IDFS; secondary outcomes include OS, distant metastasis free survival, local and axillary recurrence, and self-reported adverse events. The study, targeting Asian breast cancer patients, will also include quality of life and cost-effectiveness measures. The trial aims to recruit 1,734 patients for randomization to demonstrate 5% noninferiority margin. Results are anticipated in 2027 [31].

The VENUS trial is a prospective, noninferiority randomized controlled trial including women with cT1–2N0 invasive breast cancer < 5 cm and clinically node-negative axilla on physical exam and ultrasound [32]. Patients will be randomized to undergo SLNB or no axillary surgery, with stratification by age (≤ 50 years old and > 50 years old) and clinical tumor size (≤ 2 cm and > 2 cm). Patients may undergo either BCS or mastectomy. NAST is allowed. The primary endpoint is DFS, with secondary endpoints including OS, RRFS, axillary recurrence rate, morbidity, ultrasonography (US) accuracy, and cost effectiveness. A sample of 364 is estimated for each arm. Enrollment began in 2019, and results are anticipated in 2030 [32].

The OMSLNB (Omission of Sentinel Lymph Node Biopsy) trial is a prospective, single-arm, phase II noninferiority study including women aged 18–70 years old with invasive breast cancer ≤ 3 cm and clinically node-negative axilla confirmed on at least two imaging modalities (axillary ultrasound plus MRI, positron emission tomography–computed tomography (PET-CT), or fluorine-18 fluorodeoxyglucose (18F-FDG) PET/MRI) [33]. Patients must have an ECOG score of 0–1. Patients will undergo either BCS with WBR (excluding the axilla) or total mastectomy, without axillary surgery, with systemic therapy based on local guidelines. The primary endpoint is 3-year IDFS, with secondary endpoints including breast cancer-related lymphedema. A sample size of 311 was calculated to meet noninferiority criteria. Enrollment began in June 2023 and is expected to conclude in 2026, with results projected for 2029 [33]. Notable distinctions from prior studies include inclusion of mastectomy and focus on an Asian patient population, in addition to being a phase II open-label, single-arm clinical trial. Initial reported outcomes from these trials are summarized in Table 2 [27–29, 36].

**Table 2:** Results of Clinical Trials Evaluating Omission of Axillary Surgery in Clinically Node-Negative Early-Stage BC

Trial	Follow-up	Population	Mean age (range)	Median clinical tumor size	Lymph node positivity	Recurrence	Survival	Adjuvant therapies	Conclusion
INT09/98 [27], n = 517, enrollment: 1998–2003	20 years	Women < 65 years old with cT1N0 were randomized to quadrantectomy without axillary surgery (QU) or quadrantectomy with axillary dissection (QUAD)	52.6 years (30–65 years)	1.5 (1.1–1.9) cm	QUAD arm with 28.7%	Local recurrence (QU: 12.2%; QUAD: 12.5%) Axillary recurrence (QU: 10.6%; QUAD: 0.37%) Distant metastases (QU: 17.6%; QUAD: 13.2%)	OS (QU: 76.8%; QUAD: 77.3%; adjusted hazard ratio of QU vs QUAD arm 1.18 for OS; P = 0.326) DFS (QU: 81.5%; QUAD: 86.9%; adjusted hazard ratio of QU vs QUAD arm 1.27 for DFS; P = 0.280) BCM (QU: 14.3%; QUAD: 11%) Statistical analysis via Kaplan-Meier method, compared using log-rank test	Chemotherapy (QU: 35.5%; QUAD: 51.5%)	At 20 years follow-up, no significant difference in OS, BCM, or DFS in patients undergoing vs omitting axillary dissection
SOUND [28], n = 1,405, enrollment: 2012–2017	5 years	Women of any age with invasive BC ≤ 2 cm, cN0 by pre-op axillary US, undergoing BCS and RT (WBR or pBI), randomized 1:1 to SLNB or no axillary surgery	60 years (52–68 years)	1.1 (0.8–1.5) cm	SLNB arm with 13.7%	Local recurrence (SLNB: 1.0%; no AS: 0.9%) Axillary recurrence (SLNB: 0.4%; no AS: 0.7%) Distant metastasis (SLNB: 1.8%; no AS: 2.0%) Cumulative incidence of distant metastasis (SLNB: 2.3%; no AS: 1.9%; Gray P = 0.69) Cumulative incidence of axillary recurrences (SLNB: 0.4%; no AS: 0.4%; Gray P = 0.91)	DDFS (SLNB: 97.7%; no AS: 98.0%; P = 0.67) DFS (SLNB: 94.7%; no AS: 93.9%; P = 0.30) OS (SLNB: 98.2%; no AS: 98.4%; P = 0.72) Statistical analysis via Kaplan-Meier method, compared using log-rank test	Chemotherapy (SLNB: 20.1%; no AS: 17.5%) In HER2+ disease patients receiving trastuzumab (SLNB: 93.8%; no AS: 97.9%) RT (SLNB: 98.0%; no AS: 97.6%) pBI (SLNB: 10.7%; no AS: 10.8%) ET in ER+ BC (SLNB: 97.9%; no AS: 98.9%)	At 5 years follow-up, omission of axillary surgery was noninferior to SLNB for DDFS in postmenopausal women with ER+, HER2– BC who are cN0 with tumors ≤ 2 cm, adjuvant treatments were not significantly different between treatment groups supporting trend of decisions made based on biologic tumor data
INSEMA [29], n = 4,858, enrollment: 2015–2019	5 years	Women >18 years old with cT1–T2, cN0 BC undergoing BCS with WBR, randomized 1:4 to undergo omission of axillary surgery or to SLNB, those randomized to SLNB who were found to be pN1 were randomized 1:1 to cALND or omission of cALND	62 years (24–89 years)	1.1 (0.8–1.6) cm	SLNB arm with 15.0%	Local recurrence (SLNB 1.1%; no AS: 0.8%) Axillary recurrence (SLNB: 0.3%; no AS: 1.0%) Distant metastasis (SLNB: 2.7%; no AS: 2.7%)	5-year IDFS (SLNB: 91.7% (95% CI, 90.8–92.6); no AS: 91.9% (95% CI, 89.9–93.5)) 5-year OS (SLNB: 96.9% (95% CI, 96.3–97.5); no AS: 98.2% (95% CI, 97.1–98.9))	Chemotherapy (SLNB: 12.9%; no AS: 10.4%)	At 6 years follow-up, omission of axillary surgery was noninferior to SLNB for IDFS and suggests omission of SLNB may be reasonable for women ≥ 50 years old with HR+, HER2–, low grade, cT1 BC
BOOG2013-08 [36], n = 1,572, enrollment 2015–2022	5 years	Women ≥ 18 years old with cT1–2N0 BC undergoing BCS with WBR randomized to SLNB or no axillary surgery	61 years	Not yet reported	SLNB arm with 13.7%	Local recurrence (SLNB: 15.3%; no AS: 9.1%) Regional recurrence (SLNB: 5.1%; no AS: 9.1%) Distant metastasis (SLNB: 23.7%; no AS: 29.5%)	RRFS (SLNB: 96.6% (95% CI, 95.2–98.0); no AS: 94.2% (95% CI, 92.4–96.0)) DDFS (SLNB: 96.0% (95% CI, 94.4–97.6); no AS: 92.9% (95% CI 90.0–94.9); absolute difference of 3.3%)	Chemotherapy (SLNB: 13.0%; no AS: 11.3%) Targeted therapy (SLNB: 5.9%; no AS: 4.4%) Endocrine therapy (SLNB: 44.8%; no AS: 43.1%)	At median 5 years follow-up, omission of axillary surgery was noninferior to SLNB for RRFS and suggests omission of SLNB may be reasonable in women ≥ 50 years old with HR+, HER2–, grade 1–2, cT1 BC

BC: breast cancer; AS: axillary surgery; SLNB: sentinel lymph node biopsy; QUAD: quadrantectomy with axillary lymph node dissection; QU: quadrantectomy alone; WBR: whole breast radiation; SOUND: Sentinel Node vs Observation After Axillary Ultra-Sound; INSEMA: Intergroup Sentinel Mamma; pBI: partial breast irradiation; BOOG 2013-08: Dutch Breast Cancer Research Group 2013-08; US: ultrasonography; BCS: breast conserving surgery; IDFS: invasive disease-free survival; OS: overall survival; DFS: disease-free survival; DDFS: distant disease-free survival; RT: radiotherapy; pre-op: preoperative; cALND: completion axillary lymph node dissection; BCM: breast cancer mortality; ER: estrogen receptor; HR: hormone receptor; RRFS: regional recurrence-free survival.

As emerging data continue to support the oncologic safety of omitting SLNB in appropriately selected patients, attention has shifted toward understanding the patient experience. The next section explores the quality of life implications of foregoing SLNB.

### Impact of omitting SLNB on quality of life outcomes

Several trials evaluating the safety of omitting axillary surgery have also examined its impact on quality of life outcomes. Short-term patient-reported outcomes have been published for the INSEMA and BOOG 2013-08, while results from OMSLNB remain pending. In INSEMA, quality of life measures was compared among patients undergoing no axillary surgery, SLNB, or cALND over 18 months of follow-up [37]. Compared with omission of axillary surgery, patients in the SLNB group reported significantly greater postoperative pain, arm edema, and impaired mobility at all follow-up intervals, with the largest differences observed at 1 month. Arm swelling in the SLNB group increased over time, with pronounced differences from the omission group by 12 and 18 months. Rates of seroma formation, arm and shoulder mobility limitations, pain, and paresthesias were highest in the cALND group, intermediate in the SLNB group, and lowest in the omission group [37].

The BOOG 2013-08 trial reported 3-year arm function and quality of life outcomes among patients randomized to SLNB and no axillary surgery [38]. Questionnaires completed from baseline through 3 years demonstrated better physical arm function and mental health scores in the omission group from 6 months up to 2 years. Patients undergoing SLNB group also reported arm and breast symptoms at 6 months and 1 year; however, these differences did not reach thresholds for clinical relevance. Notably, psychological factors such as anxiety and neuroticism were more strongly associated with perceived functional impairment than surgical factors such as SLNB [38].

Overall, these studies indicate that omission of axillary surgery is associated with improved quality of life outcomes including reduced arm symptoms, better functional recovery, and lower treatment-related morbidity, supporting the selective omission of SLNB in eligible patients. These quality of life benefits, combined with comparable survival outcomes, have prompted professional societies to reconsider routine axillary staging.

## Barriers to Omission of SLNB

### Patient-driven barriers to omission of SLNB

Despite the Choosing Wisely^®^ recommendation to avoid routine SLNB in elderly, clinically node-negative, HR-positive/HER2-negative invasive breast cancer, utilization remains high. Barriers to de-implementation arise from patient-, physician-, and tumor-related factors. Patient-driven barriers include limited awareness of the guideline, poor recall of having discussed omission with their provider, a desire for reassurance, influence of friends’ and family members’ experiences, and concerns related to functional status, age, and quality of life [39]. Many patients and clinicians are not engaging in sufficiently thorough conversations to support shared decision-making, particularly when patients are unaware that de-escalation of previously standard procedures, such as SLNB, is a safe and evidence-based option in select circumstances.

Access to clear, patient-friendly information further compounds these challenges. In a systematic analysis of online resources from the top 24 cancer hospitals and four national breast cancer organizations, none of the major cancer centers and only one national organization provided readily accessible information for patients regarding omission of SLNB or adjuvant radiotherapy in older women with early-stage breast cancer [40]. This gap underscores the urgent need for accessible, comprehensible educational materials tailored to older adults, including up-to-date summaries of clinical trials and guideline recommendations, to enhance patient understanding and facilitate evidence-aligned decision-making. Patients eligible for SLNB omission frequently cite OS, BCSS, LR risk, and quality of life considerations as central to their decision-making [41]. These priorities highlight the critical role of provider–patient discussions in conveying the evidence supporting omission and in guiding shared decisions that align with patient values.

### Physician- and tumor-related barriers to omission of SLNB

A number of physician-driven barriers also contribute to the continued use of SLNB in women who are eligible for omission. These include limited awareness of the Society of Surgical Oncology (SSO) Choosing Wisely^®^ guideline, insufficient familiarity with or skepticism toward the supporting evidence, a tendency to base decisions on functional status rather than chronological age, and the perception that SLNB is a low-risk procedure [42]. Even among physicians who are aware of the guideline, some mistakenly believe it is supported solely by retrospective data, leading to hesitancy in applying it to their own patients. Additionally, although the guideline targets patients over 70 years old, some clinicians remain reluctant to omit SLNB in functionally robust older adults because they anticipate that these patients may live long enough to benefit from all available treatments, including chemotherapy.

The belief that SLNB is a quick, well-tolerated procedure with relatively low morbidity compared with ALND also influences practice. When patients request SLNB for reassurance, many physicians prioritize respect for patient autonomy. Further concerns include the perceived lack of consensus regarding specific tumor-related criteria for omission, the possibility that omitting SLNB could complicate adjuvant treatment planning for medical or radiation oncologists, and fears of malpractice liability [43].

To support the adoption of de-escalation strategies in clinical practice, multiple factors are critical: improving provider awareness of the guideline and the randomized controlled trials demonstrating the safety of omission, modeling guideline-concordant care by leaders in breast surgical oncology, creating accessible, patient-friendly educational materials, providing feedback to surgeons who continue to perform low-value care, and fostering transparent, informative discussions that support patient-centered decision-making [44].

In addition to physician-related factors, tumor-driven characteristics have also been associated with increased likelihood of undergoing SLNB despite eligibility for omission. A Surveillance, Epidemiology, and End Results (SEER)-based analysis found that lobular histology, mixed ductal-lobular histology, poor differentiation, and T2 tumors were independently associated with performance of SLNB [45]. Similarly, an NCDB study reported that higher T stage, higher grade (grade 2–3), HER2-positive disease, invasive lobular carcinoma, and invasive mammary carcinoma increased the likelihood of SLNB [46]. These tumor features confer a higher risk of nodal metastasis, which may influence clinicians’ decisions due to implications for adjuvant therapy and recurrence risk.

## Omission of SLNB After NAST With pCR in HER2+ and TNBC

The omission of SLNB following NAST represents an emerging and investigational frontier in early-stage breast cancer management. Although accumulating evidence suggests that selected patients, particularly those with HER2-positive and TNBC who achieve a pCR in the breast, may have a very low likelihood of residual nodal disease, this strategy is not yet supported by definitive randomized data and has not been adopted as standard practice. Ongoing prospective trials are actively evaluating the oncologic safety of this approach. As such, the data presented in this section should be interpreted as hypothesis-generating and reflective of a rapidly evolving field rather than current standard of care.

The primary goals of NAST include delivering systemic treatment early, reducing recurrence risk, assessing tumor responsiveness, and potentially downstaging the breast or axilla to permit less extensive surgery. Advances in neoadjuvant regimens have substantially improved rates of pCR in both the breast and the axilla, particularly in HER2-positive and TNBC. These high response rates have led to an important question: can axillary staging be safely omitted in excellent responders to NAST? Several studies have evaluated nodal response following NAST, with growing interest in whether patients with breast pCR, especially in HER2-positive and TNBC subtypes, may avoid SLNB.

A prospective single-institution cohort study from MD Anderson Cancer Center (n = 527) investigated whether patients with cT1–2N0–1, HER2-positive or TNBC who achieve breast pCR also have low risk of residual nodal disease [47]. All patients received NAST followed by standard breast and nodal surgery between 2010 and 2014. Breast pCR was defined as absence of invasive or *in situ* carcinoma; axillary pCR as absence of metastatic carcinoma. Patients presenting with cN1 were less likely to achieve breast pCR (32.5%) than those with cN0 (40.0%). Notably, all cN0 patients who achieved breast pCR were axillary pN0 (100%), with no significant difference between HER2-positive (94.7%) and TNBC (97.0%) subgroups. Among cN0 patients without breast pCR, only 5.7% had nodal disease on final pathology, compared with 57.5% of cN1 patients without breast pCR. These findings suggest that cT1N0M0 and cT2N0M0 HER2-positive and TNBC patients who achieve breast pCR represent an excellent-responder population highly unlikely to harbor axillary metastases, supporting future trials of omitting axillary surgery in combination with adjuvant WBR [47].

A large NCDB study (n = 30,821) corroborated these results [48]. In cT1–2N0–1 HER2-positive and TNBC who achieved breast pCR after NAST, breast pCR rates remained highest in HER2-positive (44.3%) and TNBC (37.2%), compared with HR-positive/HER2-negative disease (13.1%). Breast pCR was defined as no invasive disease (ypT0 or ypTis) on final pathology. In patients with cN0 HER2+ disease at presentation, 45.0% had a breast pCR and only 1.6% were ypN+. Compared to patients with TNBC who were cN0, 37.2% had breast pCR, again, only 1.6% were ypN+. Conversely, in patients with residual breast disease, nodal positivity was substantially higher,16.9% in HER2-positive and 12.6% in TNBC. Overall, the data support the findings of MD Anderson and strengthen the rationale for evaluating omission of axillary surgery in cN0 HER2-positive or TNBC who achieve breast pCR [48].

A Chinese single institution study expanded inclusion to patients with cT1–4N0–3M0 locally advanced breast cancer undergoing breast and axillary surgery between 2015 and 2019 who achieved breast pCR after NAST (n = 258) [49]. Again, breast pCR was defined as absence of invasive carcinoma or *in situ* carcinoma in the breast (ypT0 or pTis). Axillary pCR (ypN0) was defined as absence of residual carcinoma including macrometastatic and micrometastatic axillary nodal disease in axillary lymph nodes after NAST. Breast pCR was seen in only 27.1% of patients (ypT0, 18.2%; ypTis, 8.9%), likely due to the large volume of HR-positive/HER2-negative patients included in the trial. HR-positive/HER2-negative patients were less likely to get a pCR (ypT0/is) than other subtypes (P < 0.001). Among initially cN0 patients, achieving breast pCR was again associated with 100% axillary ypN0, consistent across ypT0 and ypTis subgroups. Among cN+ patients, axillary pCR was significantly more frequent in those with breast pCR (82.7%) versus those without (22.9%). The TNBC subgroup achieved an axillary pCR of 92.3%. These results further demonstrate that breast pCR strongly predicts nodal pCR, particularly in TNBC, supporting potential omission of SLNB in selected patients [49].

A pooled analysis of five studies comprising of 3,834 women with TNBC or HER2-positive disease who were initially cN0 and achieved radiologic or pathologic complete response (rCR or pCR) after NAST demonstrated a ypN+ rate of 2.16% (95% CI, 1.70–2.63), lower than the FNR typically accepted for SLNB, providing additional support for considering SLNB omission in this setting [50].

Several studies have examined predictors of breast pCR and ypN0 status following NAST. A retrospective, multicenter Chinese study of patients ≥ 18 years old from 2009 to 2020 with cT1–3N0–1 invasive breast cancer found that HER2-positive and TNBC subtypes had the highest rates of breast pCR and ypN0. Among cT1–2N0 patients who achieved breast pCR, 100% of HER2-positive and 94.1% of TNBC patients achieved ypN0, supporting omission of axillary surgery in these excellent responders [51].

A Netherlands Cancer Institute study evaluated predictors of ypN0 in cN0 patients treated with NAST [52]. The study included patients with cT1–3N0 breast cancer treated with NAST followed by breast surgery (mastectomy or lumpectomy) and SLNB between 2013 and 2018. cN0 status was determined on axillary ultrasound and PET/CT. Strong predictors of ypN0 included tumor subtype (HER2-positive and TNBC), grade III, and breast rCR on MRI. rCR occurred in 82% of HR-positive, 98% of TNBC, and 100% of HER2-positive tumors among patients who achieved ypN0. These findings later supported the initiation of the ASICS (Avoiding Sentinel lymph node biopsy In select Clinical node negative breast cancer patients after neoadjuvant Systemic therapy) trial [52].

Additional prospective and retrospective studies reinforce that HER2-positive and TNBC subtypes, HR-negative status, high grade, and achievement of breast pCR strongly predict ypN0. One prospective cohort (n = 179) including women with cN0 invasive breast cancer treated with NAST followed by surgery between 2010 and 2021 found that patients with breast pCR following NAST had 100% 5-year OS and DFS, and HER2-positive cN0 patients, Nottingham score (III) with breast pCR were particularly strong candidates for omission [53]. In a single-institution retrospective cohort study including 139 women ≥ 18 years old with HER2-positive invasive breast cancer diagnosed between 2022 and 2023, patients underwent NAST with single or dual HER2 blockage followed by BCS or mastectomy and axillary surgery to identify factors associated with breast pCR and ypN0 status [54]. Among the patients, 92.8% had suspicious axillary disease or were lymph node–positive prior to NAST; 80.6% achieved clinical and radiologically negative axillary lymph nodes; and 79.1% achieved ypN0. All patients who were cN0, had T1 tumors, or achieved radiologic nodal complete response after NAST achieved ypN0, supporting consideration of omission even in patients initially presenting with nodal disease if they convert to ycN0 [54]. Another retrospective study (n = 166) evaluated the incidence and features associated with ypN+ after NAST in cN0 invasive breast cancer [55]. The study found that ypN+ was associated with larger tumor size, higher stage, lobular histology, lymphovascular invasion, and ER-positive/HER2-negative subtype, while ypN0 strongly correlated with breast pCR and HR-negative/HER2-positive subtypes. The overall incidence of ypN+ in cN0 patients was only 11.4%. With low occult lymph node positivity, this again supports omission of SLNB in cN0 HER2+ patients who achieve breast pCR [55].

Given the accumulated evidence, several clinical trials were initiated to formally evaluate omission of SLNB in TNBC and HER2+ patients with cN0 who received NAST with excellent response in the breast. This includes EUBREAST-01 (European Breast Cancer Research Association of Surgical Trialists), ASICS, ASLAN (Avoid Axillary Sentinel Lymph Node Biopsy After Neoadjuvant Chemotherapy), and Neo-NAUTILUS trials [56–59].

The EUBREAST-01 trial is an ongoing multicenter, prospective, single-arm, non-randomized study evaluating oncologic safety of omitting SLNB in women ≥ 18 years old with cT1c–T3N0M0 TNBC or HER2-positive invasive breast cancer who achieve breast pCR after NAST [56]. cN0 status will be confirmed on physical exam and US. Recommendations for pre- and postoperative systemic therapy are based on local multidisciplinary tumor board decisions. HR-positive patients should receive postoperative endocrine therapy; those with HER2 should receive anti-HER2 treatment; and poly (adenosine diphosphate–ribose) polymerase (PARP) inhibitors or immune checkpoint-inhibitors are allowed. Adjuvant radiation with WBR with boost to the tumor bed is recommended; however, hypofractionated regimen, intensity-modulated radiotherapy, volumetric intensity-modulated arc therapy, and radiotherapy in deep inspiration breath hold are also allowed. The study discourages the use of high tangents (axillary level I and II). The primary endpoint is 3-year rate of axillary recurrence-free survival (ARFS). Secondary endpoints include 5-year invasive DFS, OS, locoregional DFS (LRDFS), DDFS, ARFS, ipsilateral axillary recurrence rate, and diagnostic accuracy of imaging methods of pCR after NAST. If the assumed acceptable 3-year ARFS ≥ 98.5%, omission of SLNB following NAST in patients with pCR would be the new recommendation based on current inclusion criteria. A 3-year ARFS ≤ 96% confirms need for SLNB, and 3-year ARFS of 96.1–98.4% indicates a need for a randomized controlled trial. The study accrual period was 24 months for an estimated need to enroll 267 patients for a power of 95%. The 3-year analysis is planned for Q4/2025, with the final analysis in Q4/2027 [56].

The ASICS trial is evaluating omission of SLNB in women ≥ 18 years of age with cT1–3N0 HER2-positive or TNBC patients who achieve rCR on MRI after NAST [57]. cN0 status is determined by US or FDG-PET/CT. The primary endpoint is axillary recurrence. Secondary endpoints include breast cancer-specific quality of life, level of cancer worry, recurrence free, overall and disease-specific survival. The 5-year outcomes will be reported after study completion. Accrual has not yet started [57].

The ASLAN (Avoid Axillary Sentinel Lymph Node Biopsy After Neoadjuvant Chemotherapy) trial is a Korean multicenter single-arm trial evaluating the oncologic safety of omitting axillary surgery in excellent responders to NAST in HER2+ and TNBC patients [58]. The study is recruiting women from five Korean tertiary care centers, who are 20–69 years of age with cT1–3N0–1M0 HER2-positive or TNBC and ECOG 0–1, expected to achieve breast pCR based on radiologic imaging. Imaging includes a combination of breast mammogram, ultrasound, and MRI. Excellent response to NAST is defined by a residual mass ≤ 2 cm on imaging or non-mass enhancement ≤ 4 cm on breast MRI, and no breast or axillary lymph node progression during NAST and physical exam after NAST. Eligible patients will undergo BCS with confirmation of breast pCR (defined as disappearance of all target lesions since baseline) before assignment of omission of SLNB. Patients will receive adjuvant radiation to the whole breast and regional lymph nodes to include axillary level I and II. Primary endpoint is 5-year RFS. Secondary endpoints include 5-year ipsilateral breast tumor recurrence interval, ipsilateral axillary recurrence interval, distant metastasis free survival, BCSS, OS, contralateral BC free survival, re-operation rate according to breast biopsy after NAST, adverse events, and quality of life. The study is ongoing with estimated completion in 2028 [58]. Results in combination with EUBREST-01 and ASICS trial are promising to influence current standards of care and potential further de-escalation of axillary surgery in excellent responders to NAST.

The Neo-NAUTILUS (No Axillary Surgical Treatment in Clinically Lymph Node Negative Patients on Ultrasonography After Neoadjuvant Chemotherapy) trial is a prospective, multicenter, randomized controlled clinical trial comparing omission of axillary surgery versus SLNB in women ≥ 20 years old with invasive breast carcinoma cT1–3N0M0 treated with NAST [60]. If pre-NAST staging is cT1–3N1M0, patient must be HER2-positive or TNBC, and have ≥ 30% reduction in tumor size on MRI after NAST. Patients must have negative axillary lymph node status on ultrasound after NAST, undergo BCS, and have an ECOG performance status of 0–2. The primary outcome is 5-year IDFS. Secondary outcomes include 1-year quality of life, and 5-year OS, distant metastasis free survival, axillary recurrence rate, and locoregional recurrence rate. Target sample size is 464 patients for a 10% noninferiority margin with an expected 5-year IDFS rate of 82% in the SLNB arm, with a one-sided significance level of 0.05 and 80% power. The study began to enroll patients in June 2025, and enrollment is expected to be complete by May 2028 [60]. This is the first randomized controlled trial evaluating omission of SLNB following NAST and expands inclusion criteria beyond pCR in the breast to those with predicted axillary response.

In conclusion, multiple prospective and retrospective studies demonstrated that cN0 HER2-positive and TNBC patients who achieve breast pCR after NAST have exceedingly low rates of residual nodal disease, supporting consideration of SLNB omission to reduce morbidity. Multiple ongoing clinical trials are evaluating this strategy in patients with cT1c–T3N0–1 HER2-positive and TNBC with complete radiologic or pathologic breast response (Table 3) [56–58, 60]. Early results, expected beginning in 2027, have the potential to change current practice and may lead to further de-escalation of axillary surgery in this subset of excellent responders to NAST. Key takeaways from this section are summarized in Table 4 [47–49, 50–52, 55–58, 60].

**Table 3:** Clinical Trials Evaluating Omission of Sentinel Lymph Node Biopsy After Neoadjuvant Systemic Therapy

Trial	Location	Study design	Population	Planned enrollment	Primary endpoint	Secondary endpoints	Axillary lymph node evaluation	Radiation
EUBREAST-01 [56] (NCT04101851)	Germany	Multicenter, prospective, single-arm trial	Women ≥ 18 years old with cT1c–3, N0, M0 TNBC or HER2+ invasive breast cancer who received NAST with rCR (by mammogram and axillary US, or MRI), undergoing BCS with WBR, will be assigned to not undergo SLNB once a pCR was confirmed in the breast (ypT0).	N = 267	3-year axillary recurrence free survival	5-year IDFS, OS, locoregional disease-free survival, ARFS, ipsilateral axillary recurrence rate, diagnostic accuracy of imaging methods	Clinical exam and pre-op axillary US prior to NAST and surgery	WBR with boost to the tumor bed, hypofractionated regimen is allowed, extension of radiation field to high tangents is not recommended
ASICS [57] (NCT04225858)	Netherlands	Prospective noninferiority cohort, single-arm trial	Women ≥ 18 years old with cT1–3, N0, M0 TNBC or HER2+ invasive breast cancer who received NAST with rCR (by MRI), will be assigned to omit SLNB.	N = 340	5-year axillary recurrence	5-year RFS, OS, DFS, and QOL outcomes	Pre-op axillary US and FDG-PET/CT	Not specified
ASLAN [58] (NCT04993625)	Korea	Multicenter, prospective, single-arm trial	Women 20–69 years old with cT1–3, N0–1, M0 HER2+ or TNBC breast cancer, expected to achieve breast pCR based on imaging (breast mammogram, US, and MRI) and physical exam following NAST. Patients will undergo BCS with adjuvant RT and omission of SLNB.	N = 178	5-year recurrence free survival	5-year ipsilateral breast tumor recurrence and ipsilateral axillary recurrence intervals, distant metastasis free survival, BCSS, OS, contralateral breast cancer free survival, re-operation rates, adverse events, and QOL	Breast mammogram, US, MRI	WBR (conventional or hypofractionated) with RNI including axillary level I and II
Neo-NAUTILUS [60] (NCT06704945)	Korea	Multicenter, prospective, randomized, controlled, phase III, noninferiority trial	Women ≥ 20 years old with cT1–3, N0, M0 breast cancer of any subtype, or select cT1-3, N1, M0 breast cancer that is HER2+ or TNBC with tumor response > 30% on MRI, undergoing BCS with adjuvant RT, randomized 1:1 to SLNB or no SLNB, were stratified by clinical nodal status and tumor subtype.	N = 464	5-year invasive disease-free survival	5-year OS, distant metastasis free survival, ipsilateral axillary recurrence, locoregional recurrence, rate of residual nodal disease, QOL outcomes	Post-NAST axillary US, breast MRI if initially cN1	WBR (conventional or hypofractionated) included either standard tangents or WBR with level I axillary coverage if cN0 in omission of SLNB arm; if cN1 WBR with standard tangents, level I axillary coverage, high tangents, or RNI

SLNB: sentinel lymph node biopsy; HER2: human epidermal growth factor receptor 2; EUBREAST: European Breast Cancer Research Association of Surgical Trialists; ASICS: Avoiding Sentinel lymph node biopsy In select Clinical node negative breast cancer patients after neoadjuvant Systemic therapy; ASLAN: Avoid Axillary Sentinel Lymph Node Biopsy After Neoadjuvant Chemotherapy; Neo-NAUTILUS: No Axillary Surgical Treatment in Clinically Lymph Node Negative Patients on Ultrasonography After Neoadjuvant Chemotherapy; TNBC: triple-negative breast cancer; MRI: magnetic resonance imaging; pCR: pathologic complete response; rCR: radiologic complete response; US: ultrasonography; BCS: breast conserving surgery; IDFS: invasive disease-free survival; OS: overall survival; DFS: disease-free survival; RFS: recurrence-free survival; RNI: regional nodal irradiation; ARFS: axillary recurrence-free survival; NAST: neoadjuvant systemic therapy; RT: radiotherapy; PET/CT: positron emission tomography/computed tomography; QOL: quality of life; WBR: whole breast radiation; pre-op: preoperative; BCSS: breast cancer-specific survival.

**Table 4:** Key Clinical Takeaways of Consideration of Omission of Sentinel Lymph Node Biopsy After Neoadjuvant Systemic Therapy

Investigational nature of SLNB omission after neoadjuvant therapy
Omission of SLNB following NAST remains investigational and is not yet standard of care.
Patient populations with the most promising data
Patients with HER2- positive and triple-negative breast cancer who achieve breast pathologic complete response demonstrate extremely low rates of residual nodal disease [47–49, 51]
In clinically node-negative patients, breast pathologic complete response is highly predictive of axillary pathologic complete response [47, 49, 51, 52]
Risk of residual nodal disease
Reported rates of residual nodal disease in clinically node-negative HER2-positive and triple-negative patients with pathologic complete response are approximately 1–2%, below accepted false-negative thresholds for SLNB [48, 50]
The likelihood of nodal involvement is higher in patients with:
Residual breast disease [47, 48]
Hormone receptor-positive/HER2-negative tumors [48, 49, 55]
Initial node-positive disease [47, 49]
Ongoing clinical trials
Multiple prospective trials are evaluating omission of SLNB in this setting, including:
EUBREAST-01 [56]
ASICS [57]
ASLAN [58]
Neo-NAUTILUS [60]
Clinical implications
If validated, omission of SLNB in carefully selected patients may:
Reduce surgical morbidity (lymphedema, pain, functional impairment)
Further advance de-escalation of axillary surgery
Support more personalized, response-adapted treatment strategies

SLNB: sentinel lymph node biopsy; HER2: human epidermal growth factor receptor 2; EUBREAST: European Breast Cancer Research Association of Surgical Trialists; ASICS: Avoiding Sentinel lymph node biopsy In select Clinical node negative breast cancer patients after neoadjuvant Systemic therapy; ASLAN: Avoid Axillary Sentinel Lymph Node Biopsy After Neoadjuvant Chemotherapy; Neo-NAUTILUS: No Axillary Surgical Treatment in Clinically Lymph Node Negative Patients on Ultrasonography After Neoadjuvant Chemotherapy.

## Future Directions

### Deimplementation strategies

Several strategies have been proposed to support omission of SLNB. These include prediction nomograms, cost-focused analyses, provider targeted de-implementation interventions, natural language models, alternative imaging approaches, and tumor genomic profiling.

Multiple institutions have developed nomograms to estimate the probability of nodal metastasis. The Memorial Sloan Kettering Cancer Center externally validated model incorporates age, tumor size, type, location, multifocality, lymphovascular invasion, and ER/PR status, achieving an area under the curve (AUC) of 0.754 [61]. University of Texas MD Anderson Cancer Center’s externally validated tool uses similar variables, in addition to triple-negative status [62]. Welsh et al [46] identified a low-risk cohort—grade 1, cT1mi–T1c (≤ 2 cm), or grade 2, clinical T1mi–T1b (≤ 1 cm)—with pathologic nodal positivity rate of 7.8%, compared to 22.3% in higher-risk patients. Importantly, this model was designed specifically for women ≥ 70 years of age with HR-positive disease. Xu et al [63] used SEER data to develop a survival-based nomogram for women in patients ≥ 70 years old with cT1–2 invasive breast cancer, identifying tumor subtype, grade, T stage, radiation, age, and marital status as independent predictors for OS and BCSS. SLNB conferred a survival benefit only in high-risk patients, supporting omission in low-risk groups [63].

Moorman et al [64] created a predictive model (AUC 0.83) using age, tumor size, grade, morphology, multifocality, and cN0 status, identifying that 32.8% of patients could safely forgo axillary surgery, with a false negative rate of 5%. Additionally, 25.8% of patients could be spared SLNB when selecting for cN0, or 26.8% when selecting for favorable tumor characteristics (smaller tumor size, no multifocality, no lobular carcinoma, and cN0) [64]. Yilmaz et al [65] developed a preoperative nomogram based on clinical and ultrasound findings to predict axillary metastasis risk and define a low-risk group eligible for omission of SLNB. The study was designed to apply to cT1–2N0 breast cancer and identify women with < 5% predicted nodal metastasis. Significant predictors of nodal positivity included clinical T stage, axillary imaging-based lymph node status, tumor location (retro-areolar vs multicentric), menopausal status, and luminal B subtype. A low-risk group for axillary metastasis (predicted < 5%) was identified including T1a/b tumors or T1c ≤ 1.7 cm, negative axillary imaging, non-retro-areolar and unicentric tumor location, with an AUC of 0.816 [65]. Wang et al similarly identified tumor features associated with < 5–10% nodal positivity among women ≥ 70 years old with ER-positive/HER2-negative, cT1–2N0M0 invasive breast cancer. Patients with cT1mi–T1b and grade 1–2 tumors had < 8% risk of nodal positivity; pT1 tumors without lymphovascular invasion had a < 10% risk of nodal positivity, and patients cT2 or pT2 without lymphovascular invasion and non-ductal/non-lobular histology had a < 5% risk of nodal positivity [66]. Collectively, nomograms may help identify candidates for omission who are not clearly low risk based on clinical factors alone.

Omitting SLNB can substantially reduce healthcare spending. Bredbeck et al reported 90-day cost savings of $4,027 per patient, translating to $322 million in annual national savings—and up to $766 million when paired with omission of adjuvant radiation [67]. Conversely, SLNB may be cost-effective in select women with longer life expectancy, good functional status, or high anxiety about forgoing axillary staging [68]. Increased costs stem from nuclear medicine mapping, anesthesia, operative time, pathology processing, and potential management of complications, all of which are important considerations during shared decision making.

Several interventions have targeted clinician behavior. Bredbeck et al identified substantial inter-facility variation in SLNB use, suggesting that targeting high-use centers may improve de-implementation [69]. Tailored messaging based on patient “maximizer/minimizer” tendencies reduced the likelihood of selecting SLNB among maximizing and neutral patients [70]. Mott et al implemented a multifaceted intervention—including geriatric assessment, preference profiling, and tailored scripts for counseling—that supported more balanced discussions, though workflow burden was a challenge [71]. An additional strategy is electronic health record “nudge” alerts, which notify surgeons when patients meeting Choosing Wisely^®^ criteria (age ≥ 70 with cT1–2N0, HR-positive/HER2-negative breast cancer). This approach has significantly reduced SLNB use while still supporting individualized decisions based on tumor biology such as size, grade, Ki-67 expression, and lower ER/PR expression profiles [72]. Emerging natural language models may further assist clinicians in identifying patients eligible for omission [73]. Together, these strategies underscore a growing collection of evidence-based patient-centered approaches that can meaningfully support appropriate omission of SLNB and advance de-implementation of low-value care in early-stage breast cancer.

### Alternatives to axillary nodal staging

In effort to reduce morbidity associated with axillary staging, an important question is how to evaluate lymph nodes noninvasively and what additional factors clinicians can incorporate into adjuvant treatment decisions. Multiple imaging modalities have been assessed for their ability to detect axillary lymph node involvement, including axillary US, MRI, and FDG PET. A metanalysis of 62 studies (30 evaluating axillary ultrasound, 10 MRI, and 24 PET) compared the sensitivity and specificity of these techniques for detecting macro- and micrometastases. Overall, axillary ultrasound demonstrated a sensitivity of 55% and specificity of 99%; MRI showed a sensitivity of 83% and specificity of 85%; and PET had a sensitivity of 49% and specificity of 94% [74]. Based on the data, the authors concluded that axillary US should remain the first-line modality for triaging nodal status given its low cost and high specificity. Axillary US has also been incorporated into several contemporary trials as part of defining clinical node-negative status when evaluating the safety of omitting SLNB.

The SOAPET (Sentinel node biopsy vs. observation after axillary PET) trial seeks to determine whether 18F-FDG dedicated lymph node positron emission tomography (LymphPET) can reliably identify node-negative patients preoperatively [75]. In stage one, clinically node-negative women aged ≥ 18 years old with invasive breast cancer and a negative axillary physical exam underwent axillary ultrasound and LymphPET, followed by SLNB or ALND. The primary endpoint was a negative predictive value (NPV) of 87.5%. The results showed comparable diagnostic accuracy between ultrasound (NPV of 86.3%) and LymphPET (NPV of 87.8%). Notably, combining the two modalities increased the NPV to 91% for micrometastases, suggesting the potential feasibility of a noninvasive approach if clinicians are willing to accept an FNR of approximately 10%. Isolated tumor cells and micrometastases were not included in the study analysis. Stage two, which omits SLNB in patients with negative LymphPET findings, is ongoing [75]. These early results may introduce a promising strategy for more accurate preoperative nodal assessment and further support efforts toward surgical de-escalation.

## Conclusions

Management of the axilla in early-stage breast cancer has undergone a major paradigm shift, driven by improved tumor biology understanding, advances in systemic therapy, and growing evidence that less invasive surgical approaches can maintain oncologic safety while reducing treatment-related morbidity. Current evidence supports omission of SLNB in carefully selected patients with clinically node-negative HR-positive/HER2-negative breast cancer when nodal status is unlikely to alter adjuvant treatment recommendations. The 2016 Society of Surgical Oncology Choosing Wisely^®^ recommendation advised against routine SLNB in women ≥ 70 years old with cN0, early-stage, HR-positive, HER2-negative invasive breast cancer treated with endocrine therapy, based on data from three randomized controlled trials and one retrospective cohort study [22–25, 76]. More recently, randomized controlled trials such as SOUND and INSEMA have demonstrated short-term oncologic safety of SLNB in patients ≥ 50 years old who are postmenopausal, with cT1N0 disease, confirmed on preoperative axillary ultrasound, HR-positive, HER2-negative, preferably ductal histology, Nottingham grade 1–2, undergoing breast-conserving surgery, with planned adjuvant breast radiation and endocrine therapy [28, 35]. These results are currently being implemented into practice and are reflected in current American Society of Clinical Oncology (ASCO) and National Comprehensive Cancer Network (NCCN) guidelines [77, 78]. Preliminary results from the BOOG2013-08 study presented at the 2025 San Antonio Breast Cancer Symposium further support this conclusion. Evidence for omission of SLNB in patients undergoing mastectomy, in those with higher-risk tumor biology such as T > 2cm, or younger patient populations remains limited and is currently under investigation in ongoing clinical trials including NAUTILUS, OMSLNB, and VENUS.

Taken together, the available evidence suggests that axillary staging is no longer universally required for all patients with early-stage breast cancer. In carefully selected patients with clinically node-negative, HR-positive/HER2-negative disease, omission of SLNB appears oncologically safe and may reduce treatment-related morbidity without compromising survival. As systemic therapies and imaging continue to improve, axillary surgery may increasingly transition from routine staging to a selective procedure reserved for patients in whom nodal information will meaningfully alter treatment decisions.

Despite growing evidence, implementation of SLNB omission in clinical practice remains inconsistent due to patient-, physician-, and tumor-related barriers. Addressing these challenges will require improved awareness of guideline recommendations, enhanced patient education, and development of clinical decision support strategies to facilitate evidence-based de-escalation.

Advances in NAST have also expanded interest in axillary de-escalation for patients with HER2-positive, and TNBCs who achieve pCR in the breast. Ongoing trials including EUBREAST-01, ASICS, ASLAN, and Neo-NAUTILUS, may further redefine axillary management in this population. As breast cancer continues to evolve toward more personalized treatment strategies, selective omission of SLNB represents an important opportunity to minimize morbidity while preserving excellent oncologic outcomes, representing an important step toward more personalized and value-based breast cancer care.

## Data Availability

The authors declare that data supporting the findings of this study are available within the article.

## Abbreviations

NSABP: National Surgical Adjuvant Breast and Bowel Project
cN0: clinically node-negative
DFS: disease-free survival
RFS: recurrence-free survival
DDFS: distant disease-free survival
OS: overall survival
BCS: breast conserving surgery
ALND: axillary lymph node dissection
SLNB: sentinel lymph node biopsy
IBCSG: International Breast Cancer Study Group
cALND: completion axillary lymph node dissection
SENTINA: SENTinel NeoAdjuvant
NAST: neoadjuvant systemic therapy
CALGB: Cancer and Leukemia Group B
ER: estrogen receptor
BCSS: breast cancer-specific survival
SSO: Society of Surgical Oncology
HR: hormone receptor
HER2: human epidermal growth factor receptor 2
pCR: pathologic complete response
TNBC: triple-negative breast cancer
QUAD: quadrantectomy with axillary lymph node dissection
QU: quadrantectomy alone
WBR: whole breast radiation
SOUND: Sentinel Node vs Observation After Axillary Ultra-Sound
INSEMA: Intergroup Sentinel Mamma
IDFS: invasive disease-free survival
RNI: regional nodal irradiation
PR: progesterone receptor
pBI: partial breast irradiation
BOOG 2013-08: Dutch Breast Cancer Research Group 2013-08
RR: regional recurrence
RRFS: regional recurrence-free survival
LR: local recurrence
NAUTILUS: No Axillary Surgical Treatment for Lymph Node-Negative Patients after Ultra-Sonography
OMSLNB: Omission of Sentinel Lymph Node Biopsy
rCR: radiologic complete response
EUBREAST: European Breast Cancer Research Association of Surgical Trialists
LRDFS: locoregional disease-free survival
ARFS: axillary recurrence-free survival
ASICS: Avoiding Sentinel lymph node biopsy In select Clinical node negative breast cancer patients after neoadjuvant Systemic therapy
ASLAN: Avoid Axillary Sentinel Lymph Node Biopsy After Neoadjuvant Chemotherapy
Neo-NAUTILUS: No Axillary Surgical Treatment in Clinically Lymph Node Negative Patients on Ultrasonography After Neoadjuvant Chemotherapy
AUC: area under the curve
SOAPET: Sentinel node biopsy vs. observation after axillary PET
